# Meta-analysis of tuberculosis incidence and risk in cancer patients treated with immune checkpoint inhibitors

**DOI:** 10.3389/fonc.2026.1723997

**Published:** 2026-03-13

**Authors:** Hui Ming, Biao Fu, Xiaogang Chen, Wenbing Hu, Hui Yu

**Affiliations:** 1 Department of Nuclear Medicine, Huangshi Central Hospital, Affiliated Hospital of Hubei Polytechnic University, Huangshi, China; 2 Department of Tuberculosis, Huangshi Central Hospital, Affiliated Hospital of Hubei Polytechnic University, Huangshi, China; 3 Department of Urology, Huangshi Central Hospital, Affiliated Hospital of Hubei Polytechnic University, Huangshi, China; 4 Department of Oncology, Huangshi Central Hospital, Affiliated Hospital of Hubei Polytechnic University, Huangshi, China

**Keywords:** cancer, immune checkpoint inhibitors, incidence, morbidity risk, tuberculosis

## Abstract

**Objective:**

To systematically evaluate the incidence of tuberculosis (TB) in cancer patients treated with immune checkpoint inhibitors (ICIs) and compare the risk of TB between ICI-treated and non-ICI-treated cancer patients, so as to provide evidence-based support for TB prevention and control in cancer patients undergoing ICI therapy.

**Methods:**

We searched the English databases including PubMed, Embase, and Cochrane Library from their inception to April 2025 for cohort studies or case-control studies that reported TB occurrence in cancer patients after ICI treatment or compared TB risk between ICI-treated and non-ICI-treated groups. Two independent researchers conducted literature screening, data extraction, and quality assessment using the Newcastle-Ottawa Scale (NOS) or Cochrane Risk of Bias Tool. Meta-analysis was performed using RevMan 5.4 and Stata 16.0 software. The pooled incidence of TB with 95% confidence interval (95%CI) in ICI-treated cancer patients was calculated; the relative risk (RR) with 95%CI was pooled to compare TB risk between the ICI and non-ICI groups. Funnel plots and Egger’s test were used to assess publication bias.

**Results:**

A total of 10 studies were included, involving 10,196 cancer patients in the ICI group and 147,528 cancer patients in the non-ICI group. Meta-analysis results showed that: ① The pooled incidence of TB in cancer patients after ICI treatment was 0.63% (95%CI: 0.47%, 0.80%); ② There was no significant difference in TB risk between the ICI group and the non-ICI group (pooled RR = 3.16, 95%CI: 0.44, 22.64, P = 0.138), with substantial heterogeneity (I²=97.1%, P<0.001). Subsequent subgroup analysis revealed that a history of TB was a critical effect modifier, with TB risk significantly elevated in the ICI group only among studies that included patients with prior TB (RR = 7.32, 95%CI:1.19, 45.12).

**Conclusion:**

Current evidence indicates that in the overall cancer population, ICI treatment was not associated with a significantly increased risk of TB compared to non-ICI regimens. Importantly, patients with a history of prior TB were found to be at significantly higher risk. This highlights the critical need for enhanced TB screening and vigilant monitoring, especially in this vulnerable subgroup, to mitigate TB-related adverse outcomes.

## 1 Introduction

In the field of cancer treatment, the emergence of immune checkpoint inhibitors (ICIs) is undoubtedly a revolutionary breakthrough. By precisely blocking immune checkpoint molecules such as programmed cell death protein 1 (PD-1), programmed death-ligand 1 (PD-L1), and cytotoxic T-lymphocyte-associated protein 4 (CTLA-4), ICIs reactivate the body’s immune system suppressed by tumors, thereby effectively recognizing and attacking tumor cells (1). This has brought the hope of long-term survival to many patients with advanced cancer. In recent years, ICIs have demonstrated excellent efficacy in the treatment of various malignant tumors, including melanoma, non-small cell lung cancer (NSCLC), renal cell carcinoma, and Hodgkin lymphoma, and have gradually become an important part of the comprehensive cancer treatment system (2, 3).

However, with the increasing clinical application of ICIs, their associated immune-related adverse events have gradually attracted great attention from clinicians. By disrupting the immune tolerance balance of the body’s immune system, ICIs not only activate anti-tumor immune responses but also may cause the immune system to attack normal tissues or organs, triggering a series of inflammatory reactions. In addition to common toxicities such as skin toxicity, gastrointestinal toxicity, and endocrine toxicity, the impact of ICIs on infection susceptibility has also become a research focus (4). Among these infections, tuberculosis (TB)—a chronic infectious disease caused by *Mycobacterium tuberculosis*—is particularly noteworthy in the context of ICI treatment (5).

TB remains a global public health challenge with high incidence and mortality. The epidemic is particularly severe in low- and middle-income countries, especially the 30 high TB burden countries that account for 87% of global incident cases (6). Cancer patients themselves have impaired immune function due to the disease, and treatment modalities such as radiotherapy and chemotherapy further weaken their immune defense capabilities, making them a high-risk group for TB (7). Since ICIs exert their effects by regulating the immune system, whether they further alter the risk of TB in cancer patients remains controversial. Some clinical observational studies have reported individual cases or small-sample data on TB occurrence in cancer patients after ICI treatment, but the results are inconsistent: some studies suggest that ICIs may increase the risk of TB (8, 9), while others find no significant association (10, 11). This heterogeneity in research results has caused great confusion in clinical practice and made it impossible to provide clear and reliable evidence-based support for TB prevention and control in cancer patients during ICI treatment (8–11).

Therefore, this study aims to comprehensively collect global studies on TB occurrence in cancer patients after ICI treatment through systematic review and meta-analysis, scientifically integrate data, accurately calculate the pooled incidence of TB in ICI-treated cancer patients, and compare it with the non-ICI group. By clarifying the impact of ICI treatment on the risk of TB in cancer patients, this study intends to provide high-quality evidence-based medical evidence for clinicians to develop strategies for TB screening, monitoring, and prevention during ICI treatment in cancer patients, ultimately ensuring patient treatment safety and improving patient prognosis.

## 2 Materials and methods

The study protocol was registered in PROSPERO (CRD420251112285) prior to data extraction to minimize bias and ensure methodological rigor.

### 2.1 Inclusion and exclusion criteria for literature

Cohort studies or case-control studies that reported TB occurrence in cancer patients after ICI treatment or compared the risk of TB between ICI-treated and non-ICI-treated cancer patients. Patients diagnosed with malignant tumors by histopathology or cytology, regardless of age, gender, tumor type, or stage. Patients must have received at least one dose of ICI treatment (e.g., PD-1 inhibitors, PD-L1 inhibitors, CTLA-4 inhibitors) or be included in the non-ICI group (e.g., receiving conventional chemotherapy, targeted therapy, surgery, or no anti-tumor treatment). Primary outcome indicators: ① Incidence of TB in the ICI-treated group; ② Comparison of TB risk between the ICI-treated and non-ICI-treated groups (expressed as RR with 95% confidence interval [95%CI]). The diagnosis of TB must comply with internationally or nationally recognized diagnostic criteria, such as comprehensive judgment based on positive *Mycobacterium tuberculosis* culture in sputum, positive acid-fast staining of sputum smears, strong positive tuberculin skin test (TST), combined with clinical symptoms and imaging findings (e.g., chest computed tomography showing typical TB lesions).

Exclusion Criteria: non-English literature; conference abstracts, case reports, reviews, animal experiments, and *in vitro* experimental studies; studies with incomplete data that cannot be used to extract or calculate key indicators such as TB incidence, RR value, and 95%CI; duplicate publications of the same data (only the latest or most comprehensive study was included).

### 2.2 Literature search strategy

The English databases PubMed, Embase, and Cochrane Library were searched from their inception to June 2025. A combination of MeSH terms (or subject terms) and free words was used for the search. The English search terms included: “Immune Checkpoint Inhibitors,” “PD-1 Inhibitors,” “Programmed Cell Death Protein 1 Inhibitors,” “PD-L1 Inhibitors,” “Programmed Death-Ligand 1 Inhibitors,” “CTLA-4 Inhibitors,” “Cytotoxic T-Lymphocyte-Associated Protein 4 Inhibitors,” “nivolumab,” “pembrolizumab,” “durvalumab,” “avelumab,” “atezolizumab,” “ipilimumab,” “tremelimumab,” “cemiplimab,” “camrelizumab,” “sintilimab,” “tislelizumab,” “toripalimab,” “tuberculosis,” “tuberculoses,” “Kochs Disease,” “Koch’s Disease,” “Koch Disease.” The detailed search strategies for each database are provided in **Supplementary Table 1**. Additionally, the reference lists of included studies were manually searched to supplement relevant studies that might have been missed.

### 2.3 Literature screening and data extraction

Two researchers trained systematically independently conducted literature screening according to the pre-defined inclusion and exclusion criteria. First, the titles and abstracts of the literature were read to preliminarily exclude studies that clearly did not meet the inclusion criteria. For studies that might meet the inclusion criteria, the full texts were further obtained, carefully read, and evaluated to determine whether they should be finally included. In case of disagreements between the two researchers during the screening process, consensus was reached through discussion or consultation with a third senior researcher. After literature screening, a pre-designed data extraction form was used to extract key information from the included studies. The extracted content included: basic study information: first author, publication year, country/region, and study type; characteristics of the study population: sample size (separate sample sizes for the ICI and non-ICI groups), tumor type, type of ICI and treatment regimen, patient age, gender distribution, and follow-up duration; data related to outcome indicators: number of TB cases in the ICI group and non-ICI group, or raw data that could be used to calculate TB incidence, RR value, and 95%CI; indicators related to quality assessment: For cohort studies, information such as whether random sampling was used, whether confounding factors were adjusted, and follow-up rate; for case-control studies, information such as matching between the case group and control group, and measurement method of exposure factors. data extraction was independently completed by two researchers, and cross-verification was conducted after extraction. In case of discrepancies, the original texts were reviewed and resolved through discussion.

### 2.4 Literature quality assessment

The quality of the included studies was assessed using the Newcastle-Ottawa Scale (NOS) for case-control and cohort studies. This instrument assesses the potential for bias due to confounding, in selection of participants, in measurement of interventions, due to departures from intended interventions, due to missing data, and in measurement of outcomes. The total score was 9, with a score ≥6 indicating high-quality literature. Two researchers independently assessed the quality, and disagreements were resolved through discussion. A higher score indicates a lower risk of bias.

### 2.5 Statistical analysis

We employed both fixed-effects and random-effects models based on the degree of heterogeneity. Following conventional benchmarks where I² values of 25%, 50%, and 75% represent low, moderate, and high heterogeneity, respectively by Higgins et al. (12), we used the DerSimonian-Laird random-effects model when I² ≥ 50% or when the Q-test p-value was < 0.10. And the source of heterogeneity was further analyzed. Publication bias was assessed using Egger’s regression test. *P* ≥ 0.05 or a 95% CI including 0 indicated no publication bias. *P* < 0.05 was considered statistically significant. All analyses were performed using Stata 16.0, with the ‘metan’ package for meta-analyses. The pooled TB incidence was derived using a random-effects meta-analysis of proportions with Freeman-Tukey transformation.

### 2.6 Ethics statement

This systematic review utilized only publicly available aggregated data from published studies and did not involve access to individual patient records. Therefore, ethical approval was waived by our institutional review board in accordance with the Helsinki Declaration guidelines for secondary research.

## 3 Results

### 3.1 Literature search results

**Figure 1:**
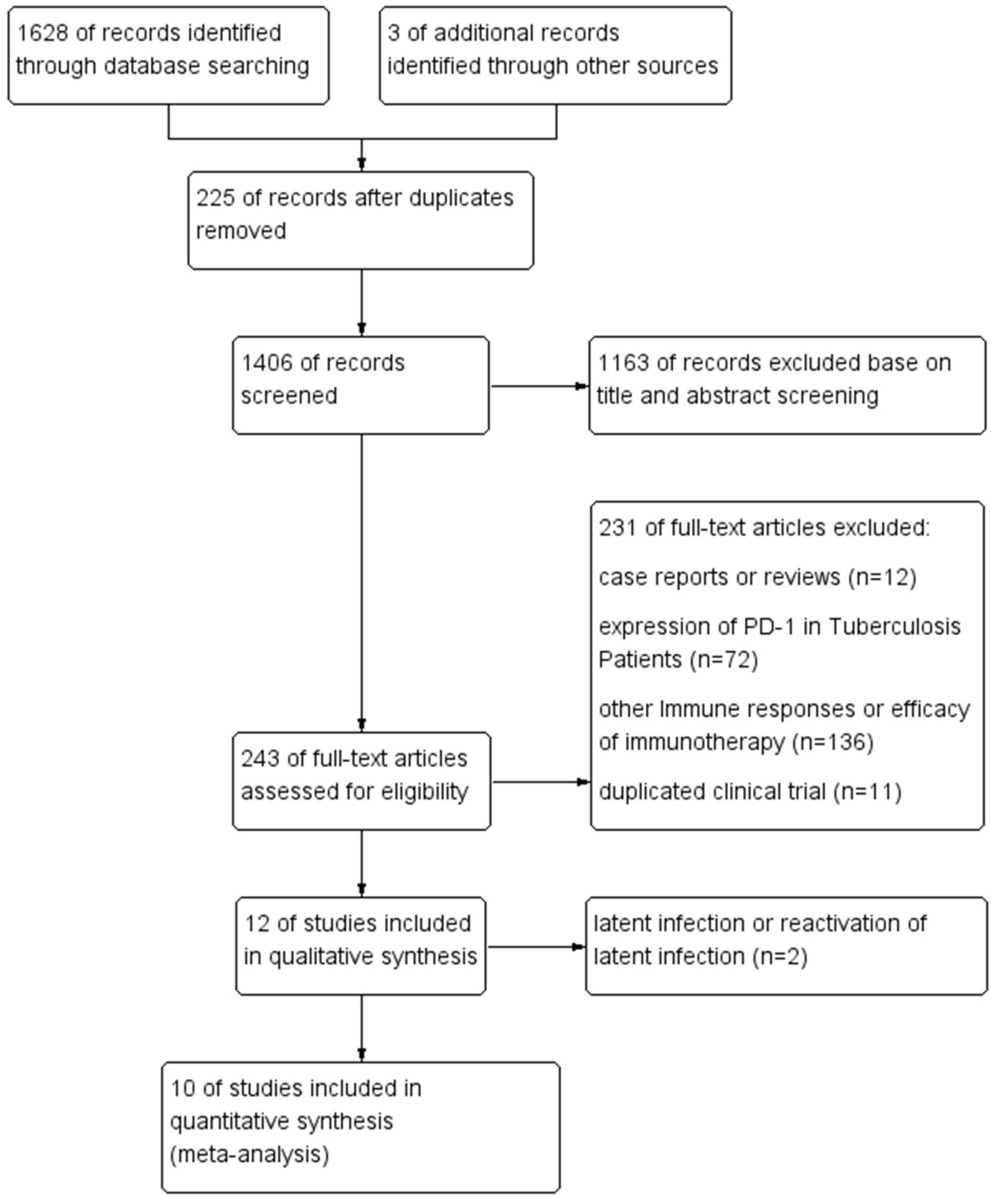
Flow diagram of included studies.

A total of 1,631 relevant studies were initially retrieved from databases and manual searches. After removing 225 duplicate studies using EndNote software, 1,406 studies were screened based on titles and abstracts. A total of 1,163 studies that clearly did not meet the inclusion criteria (e.g., conference abstracts, reviews, animal experiments, and studies with non-cancer patients as the research population) were excluded, leaving 243 studies for full-text evaluation. After applying the predefined inclusion and exclusion criteria and excluding studies with abnormal data or incomplete information, 12 studies were eventually included for qualitative analysis. After excluding 2 studies (13, 14) on diagnostic testing for latent tuberculosis infection (LTBI), 10 studies (8, 9, 11, 15–21) were included for meta-analysis. The flowchart of literature search and results is shown in **Figure 1**.

### 3.2 Basic characteristics and quality assessment of included studies

A total of 10 studies were included, among which 8 were retrospective studies and 2 were prospective studies. The study regions included Japan, South Korea, China (including Taiwan region), etc. The studies involved various tumor types treated with ICIs, including lung cancer (8,437 cases), urothelial carcinoma (337 cases), melanoma (827 cases), and esophageal squamous cell carcinoma (326 cases), with some studies focusing on multiple tumor types. The treatment regimens in the ICI group included ICIs alone, ICI combined with platinum-based chemotherapy, and PD-1 inhibitor combined with chemotherapy. Among the 10 studies, 5 only included the ICI group without a control group, and the other 5 used non-ICI treatment as the control group. The treatment regimens in the control group included tyrosine kinase inhibitors (TKIs), conventional chemotherapy, platinum-based chemotherapy, and placebo combined with chemotherapy. The total sample size of the ICI group was 10,196 (with 87 TB cases), and the total sample size of the non-ICI group was 147,528 (with 999 TB cases).

Quality assessment using the NOS showed that 9 studies were high-quality (score 8–9) and one study was moderate-quality (score 6), with no low-quality studies. The basic characteristics of the included studies are shown in **Table 1**, and the quality assessment results are shown in **Table 2**.

**Table 1:** Characteristics of included studies.

Year	Author	Type of study	Region	Tumor	Prior TB was excluded	ICI group			Non-ICI group		
						Treatment	Patients, n	TB cases	Treatment	Patients, n	TB cases
2020	Im (9)	retrospective study	South Korea	cancers	No	PD-1/PD-L1	1144	3	/	/	/
2020	Fujita (15)	retrospective study	Japan	lung cancer	No	PD-1/PD-L1	297	5	/	/	/
2020	Chan (11)	retrospective study	Singapore	lung cancer	No	ICIs	191	12	/	/	/
2022	Fujita (18)	prospective study	Japan	lung cancer	No	PD-1/PD-L1	123	2	/	/	/
2025	Wang (21)	retrospective study	China	lung cancer	No	PD-1/PD-L1	710	13	/	/	/
2021	Kim (17)	retrospective study	South Korea	lung cancer	No	ICI+chemotherapy	899	15	chemotherapy	5436	63
2021	Bae (16)	retrospective study	South Korea	lung cancer, urothelial carcinoma, or melanoma	Yes	PD-1/PD-L1	5073	20	non-ICI	136513	916
2023	Xu (19)	prospective study	Multiple countries	oesophageal cancer	No	PD1+chemotherapy	326	1	placebo+chemotherapy	326	0
2024	Chen (20)	retrospective study	China	lung cancer	Yes	ICIs	442	7	TKIs	1607	11
2025	Park (8)	retrospective study	South Korea	lung cancer	No	PD-1/PD-L1	991	9	chemotherapy	3646	9

ICIs, Immune Checkpoint Inhibitors; TB, Tuberculosis; TKIs, Tyrosine Kinase Inhibitors.

**Table 2:** Quality evaluation.

First author	Type of study	Selection	Comparability	Outcome	Scoring
Im (9)	Cohort Study	3	0	3	6
Fujita (15)	Cohort Study	4	1	3	8
Chan (11)	Cohort Study	4	1	3	8
Fujita (18)	Cohort Study	4	1	3	8
Wang (21)	Cohort Study	3	2	3	8
Kim (17)	Cohort Study	4	2	3	9
Bae (16)	Cohort Study	4	2	3	9
Xu (19)	Cohort Study	4	2	3	9
Chen (20)	Cohort Study	4	2	3	9
Park (8)	Cohort Study	4	2	3	9

### 3.3 Meta-analysis results

#### 3.3.1 Incidence of TB in cancer patients after ICI treatment

**Figure 2:**
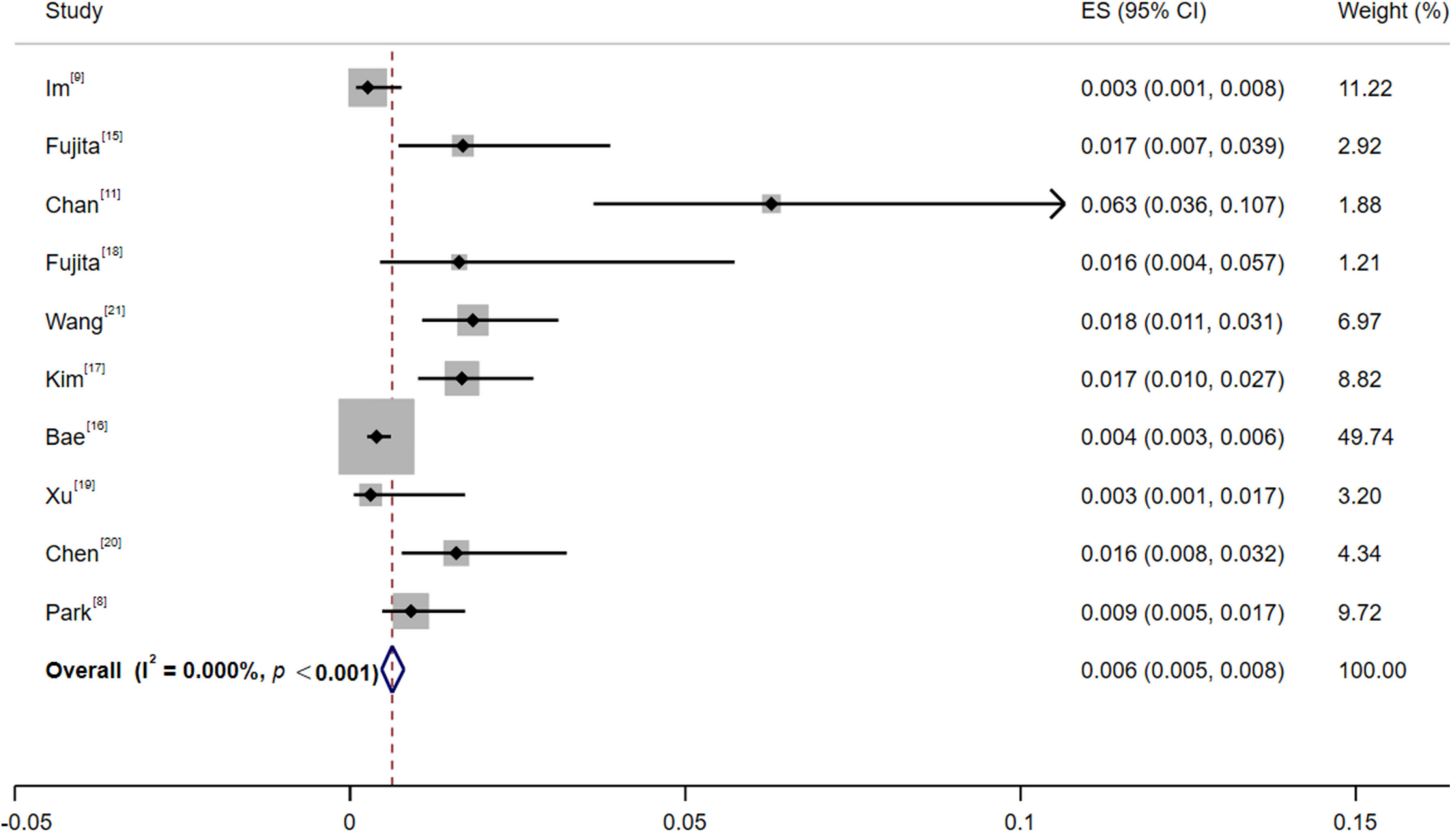
Forest plot of tuberculosis incidence in cancer patients after ICI treatment. A fixed-effects model was used. ES represents the incidence of TB in cancer patients treated with ICIs.

Among the 10 included studies (8, 9, 11, 15–21), the ICI group included 10,196 patients with 87 TB cases. Seven studies specified the type of ICI, while the other 3 did not define the ICI type. Two studies excluded patients with a previous history of TB, while the other 8 did not clearly state this information. Assessment of heterogeneity showed a statistically significant but quantitatively negligible degree of heterogeneity (I² < 0.1, P < 0.001). The significant P-value is likely driven by the large overall sample size, while the extremely low I² value suggests that the variation across studies is clinically unimportant. In accordance with methodological guidelines that prioritize the I² statistic for interpreting heterogeneity magnitude, a fixed-effects model was deemed appropriate for pooling the data. The results showed that the pooled incidence of TB in cancer patients after ICI treatment was 0.63% (Z = 12.34, 95%CI: 0.47%~ 0.80%) (**Figure 2**).

#### 3.3.2 Comparison of TB risk between the ICI group and control group

Among the 5 studies (8, 16, 17, 19, 20) that included a control group, the ICI group had 7,731 patients (52 TB cases) and the control group had 147,528 patients (999 TB cases). The heterogeneity test showed significant statistical heterogeneity among the studies (P<0.001, I²=97.1%), so a random-effects model was used for meta-analysis. The results showed that there was no significant difference in TB risk between the ICI group and the non-ICI group (pooled RR = 3.16, 95%CI: 0.44~ 22.64, P = 0.138).

**Figure 3:**
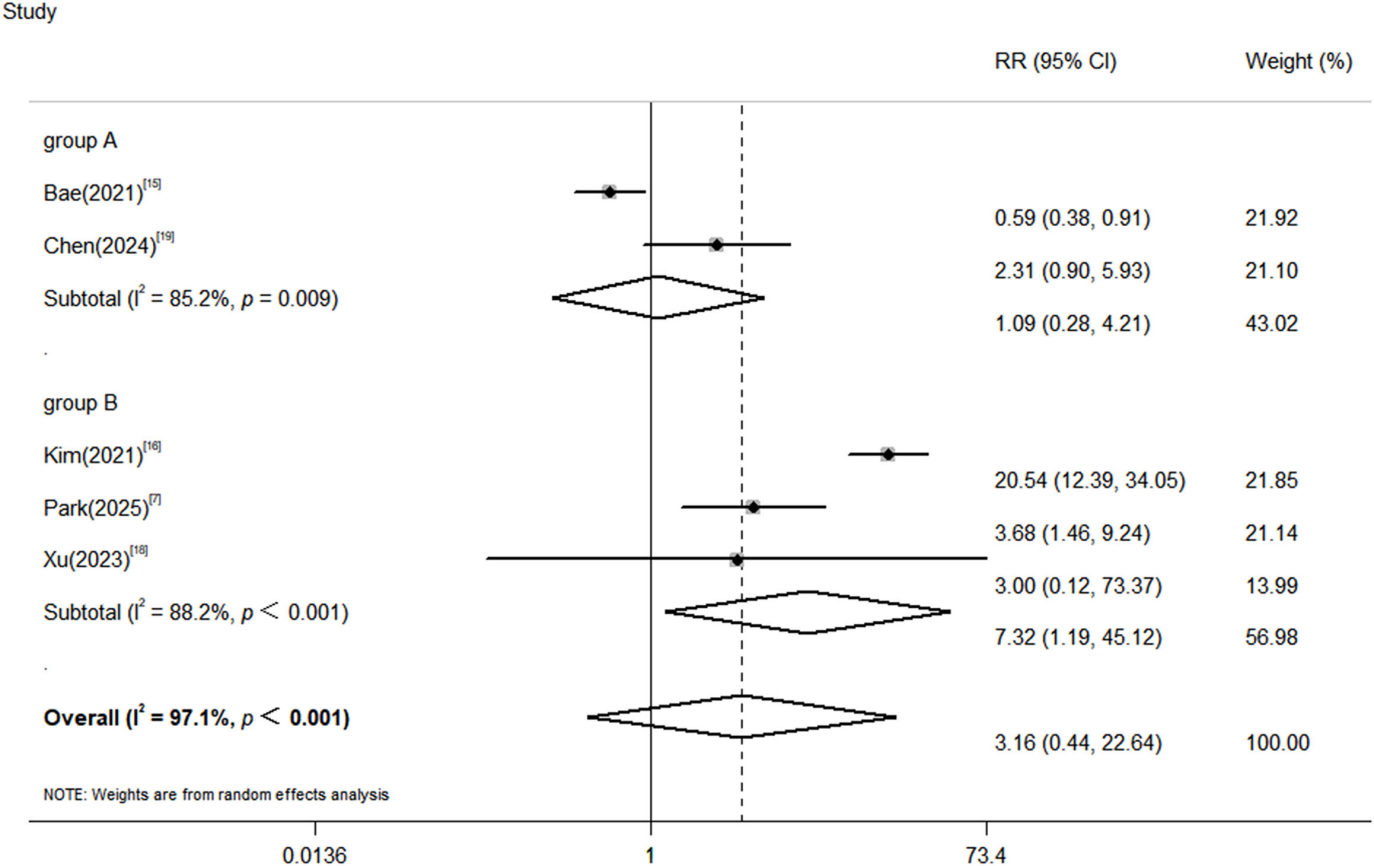
Forest plot of comparison of tuberculosis risk between the ICI group and non-ICI group. Group A: Studies with explicit exclusion of prior TB (n=2). Group B: Studies without such exclusion or with an unknown proportion of prior TB (n=3).

Further subgroup analysis was conducted based on whether patients with a previous history of TB were excluded (**Supplementary Table 2**). The results showed that in the subgroup excluding patients with a previous history of TB (2 studies), there was no statistical difference in TB risk between the ICI group and the non-ICI group. However, in the subgroup not excluding patients with a previous history of TB (3 studies), the risk of TB in the ICI group was significantly higher than that in the non-ICI group (pooled RR = 7.32, 95%CI:1.19~ 45.12, P<0.001). It is noteworthy that the 95% CI for this subgroup estimate is particularly wide, reflecting the uncertainty inherent in this finding, likely due to the limited number of studies (n=3) and patients contributing to this specific analysis. This may be the reason for the high heterogeneity among the studies (**Figure 3**). Therefore, although this result strongly suggests that a prior history of TB is a key risk modifier, the exact magnitude of the relative risk (RR) still needs to be confirmed in larger prospective studies.

### 3.4 Publication bias

#### 3.4.1 Publication bias for TB incidence

Egger’s test was used to assess publication bias for the incidence of TB in cancer patients after ICI treatment (**Supplementary Figure 1A**). The results showed that the bias term was statistically significant (P = 0.034), indicating the presence of publication bias, which may be related to the higher likelihood of publishing studies with positive results. To further verify the impact of bias on the results, the Trim and Fill method was used for adjustment. The trimming and filling analysis (**Supplementary Figure 1B**) showed that the iterative process converged (finally trimming 5 studies and including 15 studies after filling). The heterogeneity test indicated no statistical heterogeneity among the studies (P = 1.000), so a fixed-effects model was used for pooling effect sizes. After adjusting for publication bias, the pooled incidence of TB was 0.5% (95%CI: -1.3% to 2.3%, P = 0.576), which was still not statistically significant. This indicates that although publication bias exists, the conclusion of this study on “the incidence of TB in cancer patients after ICI treatment” is robust, i.e., the bias did not have a substantial impact on the non-statistical significance of the results.

#### 3.4.2 Publication bias for TB risk comparison

To evaluate potential publication bias regarding the comparison of TB risk between the ICI and non-ICI groups, we employed both funnel plot visualization and Egger’s linear regression test.

Funnel plot analysis was performed with the RR plotted on the horizontal axis and the standard error of the log-transformed RR on the vertical axis, along with a pseudo 95% CI. Despite the limited number of included studies (n = 5), resulting in only four points on the left and one on the right, no clear unilateral gap or skewed clustering was observed. The study points were distributed approximately symmetrically around the pooled effect estimate, suggesting a low risk of publication bias (**Supplementary Figure 2**).

Egger’s test was conducted using linear regression, where the standardized effect size served as the independent variable and the standard error of the effect size as the dependent variable. The intercept was not statistically significant (t =–0.21, P = 0.851; 95% CI: –113.72 to 99.95), further supporting the absence of substantial publication bias.

In summary, based on the roughly symmetric funnel plot and the non-significant Egger’s test result, there is currently insufficient evidence to indicate publication bias in the analysis of TB risk between ICI and non-ICI groups. However, it should be noted that the small number of studies may limit the statistical power of these tests. If more studies become available in the future, re-evaluation of publication bias is recommended to verify the robustness of the findings.

### 3.5 Sensitivity analysis

Sensitivity analysis was conducted to assess the robustness of the meta-analysis results regarding the incidence of TB in cancer patients following ICI treatment and the comparative TB risk between ICI and non-ICI groups. This was performed by systematically excluding each included study one at a time and recalculating the pooled estimates (**Supplementary Figure 3**). The results demonstrated that omitting any individual study did not substantially alter the overall findings. The recalculated 95% CI for the pooled TB incidence remained within the range of 0.0047 to 0.0080 (**Supplementary Figure 3A**), while that for the pooled RR consistently included values between 0.44 and 22.64 (**Supplementary Figure 3B**). All P-values exceeded 0.05, aligning with the original analysis. These findings suggest that the results are robust and that no single study disproportionately influenced the overall conclusions.

## 4 Discussion

Cancer patients already exhibit a higher baseline incidence of TB compared to the general population due to underlying immune impairment. This study systematically reviewed and analyzed evidence, predominantly from Asian cohorts as reported in the English-language literature, on TB occurrence in cancer patients following ICI treatment. By including 10 cohort studies (9 of high quality and 1 of moderate quality), we evaluated the impact of ICI therapy on TB incidence and risk. The findings indicate that although ICI treatment provides significant survival benefits for cancer patients, the risk of TB development during therapy remains a concern.

In recent years, multiple studies have investigated the relationship between ICI treatment and infectious complications, including TB, in cancer patients, yet findings have been inconsistent. For example, a retrospective cohort study (8) from South Korea involving 899 cancer patients reported TB incidence rates of 2582.5 per 100,000 person-years in the ICI group and 2108.5 per 100,000 person-years in the conventional chemotherapy group, concluding that ICI treatment did not significantly increase TB risk. In contrast, a study from China (20) indicated a higher TB incidence in lung cancer patients treated with ICIs compared to those receiving TKIs (2298 vs. 412 per 100,000 person-years, P = 0.0165). In this study, we found that when compared to non-ICI treatments (such as conventional chemotherapy or targeted therapy), no statistically significant difference in TB risk was observed in the ICI group (pooled RR = 3.16, 95% CI: 0.44~22.64, P = 0.138). This result contrasts with some previous single-center, small-sample studies (5, 22), which may be explained by the larger sample size and enhanced statistical power achieved through this meta-analysis, providing a more objective assessment of the association between ICI treatment and TB risk.

The observed discrepancies in TB incidence among ICI-treated patients across studies may be attributed to multiple interrelated factors. First, geographical disparities in TB prevalence are significant. High-burden regions including South-East Asia (34% of global cases), Africa (25%) and the Western Pacific (27%) have substantially higher TB incidence rates compared to Europe (1.9%) and the Americas (3.3%) (6). Such differences in background risk may affect the detectable association between ICI treatment and TB reactivation (8). Beyond geographical factors, heterogeneity in patient characteristics represents another important consideration. Variations in tumor types, disease stages, and baseline immune status may substantially modify both ICI therapeutic effects and susceptibility to opportunistic infections (16). For instance, patients with hematological malignancies or advanced solid tumors often exhibit more profound immunosuppression, which could interact differently with ICI-mediated immune modulation (23). Pharmacovigilance data underscore specific high-risk demographics. For instance, an analysis of the FDA Adverse Event Reporting System (24) indicated that among reported cases of PD-1/PD-L1 inhibitor-associated TB, lung cancer was the most common indication (61.11%), and patients were predominantly male (80.32%) with a median age of 68.5 years. This pattern is corroborated by clinical cohort data. A retrospective analysis by Shi et al. (10) likewise reported that ICI-associated TB cases were concentrated among males, individuals of Asian descent, and patients with advanced lung cancer. Furthermore, differences in ICI treatment regimens may contribute to inconsistent findings. Emerging evidence suggests that various classes of ICIs (e.g., PD-1 inhibitors, PD-L1 inhibitors, and CTLA-4 inhibitors) may differentially affect TB-specific immune responses. Combination immunotherapies could also amplify such effects through synergistic immune activation, though current evidence remains limited (25). Methodological differences across studies represent another layer of complexity. Methodological variations across studies, including differences in follow-up duration (11, 17) and diagnostic protocols (14), may further influence TB case ascertainment and reported incidence rates.

While ICI therapy was not associated with a significantly increased risk of TB in an unselected cancer population, our subgroup analysis indicated a notably higher risk among those with a prior history of TB. This underscores the necessity for vigilant screening and monitoring in this specific high-risk subgroup. The risk of TB reactivation is consistently present during ICI therapy and a positive interferon-gamma release assay (IGRA) result before ICI administration or IGRA conversion during ICI therapy may predict the development of active TB in these patients (18). This risk is particularly pronounced in patients with a history of TB. Zhang et al. (26) observed TB reactivation in 3 out of 40 (7.5%) NSCLC patients with a history of pulmonary TB following PD-1 inhibitor therapy, necessitating interruption of cancer treatment for anti-TB therapy. Their study further emphasized that patients with a prior TB episode carry a significantly higher risk for active TB development after anti-PD-1 treatment compared to those with latent TB infection. Consistent with these findings, a large-scale real-world observational study by Zhan et al. (27) involving 8,199 cancer patients receiving ICI therapy also identified pre-existing TB as a robust risk factor for TB occurrence following ICI initiation (OR 3.277; 95% CI, 1.822–5.895; p<0.001). Their study, conducted in a high TB-burden setting, demonstrated that the incidence of TB after ICI therapy was notably higher in individuals with a prior TB diagnosis, reinforcing the notion that ICI-induced immune modulation may precipitate the reactivation of latent or inadequately treated TB. Therefore, a history of active TB represents a potent, identifiable risk factor that demands a distinct management strategy.

Therefore, all patients initiating ICI therapy should undergo a comprehensive TB risk assessment, which must include a detailed history specifically inquiring about prior TB disease or treatment. For patients with a positive TB history, a heightened screening protocol is warranted, typically combining an IGRA or TST with baseline chest CT imaging to evaluate for old, potentially unstable lesions (5, 10, 13, 18). For high-risk groups, specifically those with a history of TB (inactive/healed) or LTBI, clinicians should strongly consider evaluating for preventive therapy. The decision should be individualized, weighing the patient’s immune status, radiological findings (e.g., fibrotic scars), local TB prevalence, and the anticipated benefits of ICI (10, 14). If active TB is diagnosed, prompt initiation of standard anti-TB therapy is necessary. ICI initiation should generally be postponed until a period of effective TB control is achieved, balancing oncology and infectious disease priorities (13). Reassuringly, as the pharmacovigilance data from Anand et al. (24), show that successful resumption of ICI after TB control is possible, offering crucial clinical reassurance that TB reactivation is a manageable complication, not an absolute contraindication to life-saving immunotherapy.

Despite adhering to established methodological guidelines, this study has limitations. The small number of included studies, particularly for the ICI versus non-ICI comparison (n=5), limits the statistical power and precision of our estimates. This is most evident in the critical subgroup of patients with a history of TB, where the effect estimate is highly imprecise (RR = 7.32, 95% CI: 1.19–45.12). While suggesting an increased risk, the wide confidence interval precludes a definitive quantification of the risk magnitude. Assessment of publication bias for TB incidence indicated statistically significant bias. Although the Trim and Fill adjustment did not alter the non-significant results, suggesting some robustness, the potential influence of unpublished negative studies cannot be entirely excluded. Heterogeneity across studies—due to variations in regional TB prevalence, healthcare standards, ICI regimens, and follow-up durations—may also affect the pooled results. The lack of detailed clinical data in some studies limited further subgroup analysis and in-depth interpretation. Lastly, our search was limited to English publications, and the included studies were predominantly from Asia; therefore, the findings may not be fully generalizable to other geographic or healthcare settings.

To address these limitations and advance the field, future research should focus on (1): conducting multicenter, large-scale prospective cohort studies with long-term follow-up to validate the association between ICI treatment and TB risk, with comprehensive collection of clinical and treatment data (2); specifically recruiting and analyzing sufficient numbers of cancer patients with a prior history of TB to generate more precise estimates of TB reactivation risk following ICI therapy, thereby narrowing the currently wide confidence intervals and enabling better risk stratification; and (3) investigating high-risk populations—such as those from TB-endemic regions, individuals with LTBI, or immunocompromised patients—to clarify specific TB risks and optimize preventive strategies.

## 5 Conclusion

Current evidence indicates that cancer patients treated with ICIs have a certain risk of developing TB, with a pooled incidence of 0.63% (95%CI: 0.47%, 0.80%). In conclusion, compared with non-ICI treatment, ICI therapy was not associated with a significantly increased overall risk of TB in the general cancer population. However, patients with a history of TB constitute a high-risk subgroup in whom the risk appears significantly elevated. These findings advocate for rigorous pre-treatment screening and targeted monitoring, particularly in this vulnerable subgroup.

## Data Availability

The original contributions presented in the study are included in the article/**Supplementary Material**. Further inquiries can be directed to the corresponding authors.
