# Correction: Meta-analysis of tuberculosis incidence and risk in cancer patients treated with immune checkpoint inhibitors

**DOI:** 10.3389/fonc.2026.1866448

**Published:** 2026-05-04

**Authors:** Hui Ming, Biao Fu, Xiaogang Chen, Wenbing Hu, Hui Yu

**Affiliations:** 1 Department of Nuclear Medicine, Huangshi Central Hospital, Affiliated Hospital of Hubei Polytechnic University, Huangshi, China; 2 Department of Tuberculosis, Huangshi Central Hospital, Affiliated Hospital of Hubei Polytechnic University, Huangshi, China; 3 Department of Urology, Huangshi Central Hospital, Affiliated Hospital of Hubei Polytechnic University, Huangshi, China; 4 Department of Oncology, Huangshi Central Hospital, Affiliated Hospital of Hubei Polytechnic University, Huangshi, China

**Keywords:** cancer, immune checkpoint inhibitors, incidence, morbidity risk, tuberculosis

An incorrect number was provided for “Grant No. 2026AFD0055”. The correct number is “Grant No. 2026AFC0055”.

The original version of this article has been updated.

