# Supplementary material for: Meta-analysis of tuberculosis incidence and risk in cancer patients treated with immune checkpoint inhibitors
Source: Front Oncol. 2026 Mar 13;16:1723997. doi: 10.3389/fonc.2026.1723997 (PMC13021448; doi:10.3389/fonc.2026.1723997)
Supplement: Supplementary file 1 [file DataSheet1.doc]

Supplement Figures and Tables

Figure S1. Funnel plots assessing publication bias for tuberculosis incidence: (A) primary meta-analysis, (B) Trim and Fill-adjusted analysis


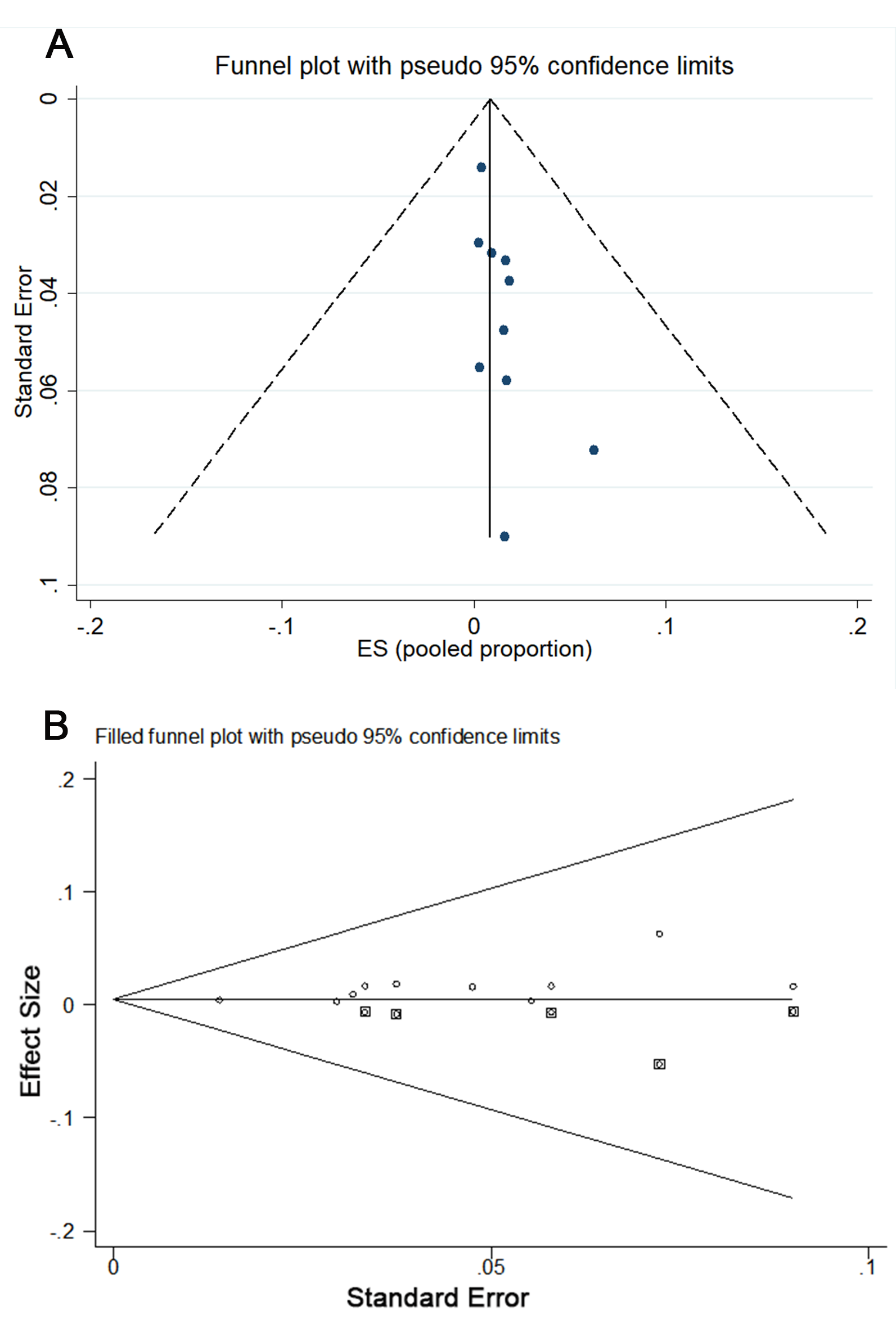


Note:The effect size (ES) on the horizontal axis is the Freeman-Tukey double arcsine transformed incidence proportion of tuberculosis for each study.

Figure S2. Funnel plot assessing publication bias for tuberculosis risk in ICI-treated versus non-ICI patients


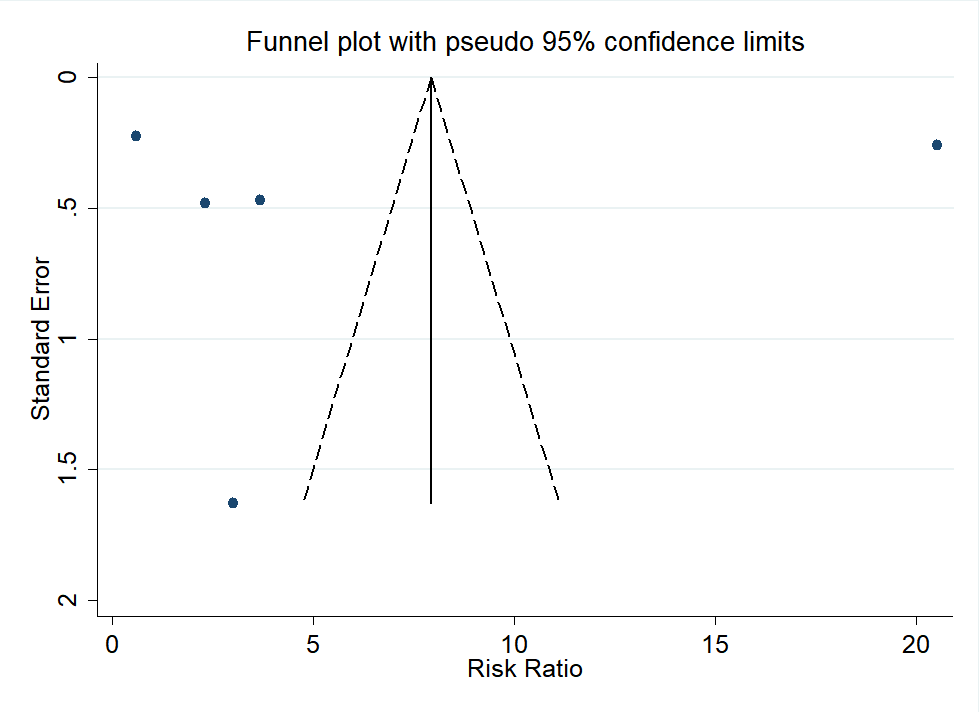


Figure S3. Sensitivity analysis: (A) TB incidence, (B) relative risk


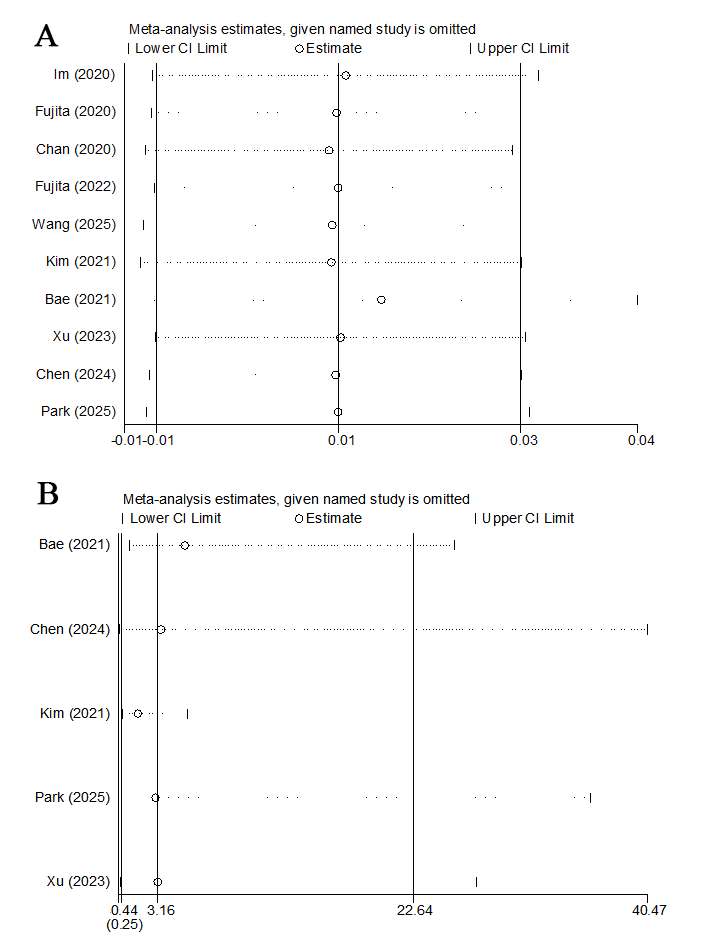


Table S1: Search methods

| Database | Steps | Search methods |
| --- | --- | --- |
| PubMed | #1 | (((((((((((((((((((((((((((((((((((((((((((((Immune Checkpoint Inhibitors[MeSH Terms]) OR (Checkpoint Inhibitors, Immune)) OR (Immune Checkpoint Inhibitor)) OR (Checkpoint Inhibitor, Immune)) OR (Immune Checkpoint Blockers)) OR (Checkpoint Blockers, Immune)) OR (Immune Checkpoint Blockade)) OR (Checkpoint Blockade, Immune)) OR (Immune Checkpoint Inhibition)) OR (Checkpoint Inhibition, Immune)) OR (PD-L1 Inhibitors)) OR (PD L1 Inhibitors)) OR (PD-L1 Inhibitor)) OR (PD L1 Inhibitor)) OR (Programmed Death-Ligand 1 Inhibitors)) OR (Programmed Death Ligand 1 Inhibitors)) OR (PD-1-PD-L1 Blockade)) OR (Blockade, PD-1-PD-L1)) OR (PD 1 PD L1 Blockade)) OR (CTLA-4 Inhibitors)) OR (CTLA 4 Inhibitors)) OR (CTLA-4 Inhibitor)) OR (CTLA 4 Inhibitor)) OR (Cytotoxic T-Lymphocyte-Associated Protein 4 Inhibitors)) OR (Cytotoxic T Lymphocyte Associated Protein 4 Inhibitors)) OR (Cytotoxic T-Lymphocyte-Associated Protein 4 Inhibitor)) OR (Cytotoxic T Lymphocyte Associated Protein 4 Inhibitor)) OR (PD-1 Inhibitors)) OR (PD 1 Inhibitors)) OR (PD-1 Inhibitor)) OR (Inhibitor, PD-1)) OR (PD 1 Inhibitor)) OR (Programmed Cell Death Protein 1 Inhibitor)) OR (Programmed Cell Death Protein 1 Inhibitors)) OR (Nivolumab)) OR (Pembrolizumab)) OR (Durvalumab)) OR (Avelumab)) OR (Atezolizumab)) OR (Ipilimumab)) OR (Tremelimumab)) OR (Cemiplimab)) OR (Camrelizumab)) OR (Sintilimab)) OR (Tislelizumab)) OR (Toripalimab) |
|  | #2 | ((((((((Tuberculosis[MeSH Terms]) OR (Tuberculoses)) OR (Mycobacterium tuberculosis Infection)) OR (Infection, Mycobacterium tuberculosis)) OR (Infections, Mycobacterium tuberculosis)) OR (Mycobacterium tuberculosis Infections)) OR (Kochs Disease)) OR (Koch's Disease)) OR (Koch Disease) |
|  | #3 | ^#^1 AND ^#^2 |
|  | #4 | ^#^3 Filters: human |
| Embase | #1 | 'immune checkpoint inhibitor'/exp OR 'immune checkpoint inhibitor'' OR 'immune checkpoint blocker' OR 'immune checkpoint inhibitors' OR 'immune checkpoint inhibitor' OR 'programmed death 1 receptor' OR 'antigen CD279' OR 'CD279 antigen' OR 'PD 1 protein' OR 'PDCD1 protein' OR 'programmed cell death 1 protein' OR 'programmed cell death 1 receptor' OR 'programmed cell death protein 1' OR 'programmed death 1 protein' OR 'programmed death protein 1' OR 'protein PD 1' OR 'protein PDCD1' OR 'protein programmed cell death 1' OR 'protein programmed death 1' OR 'programmed death 1 receptor' OR 'programmed death 1 ligand 1' OR 'antigen B7 H1' OR 'antigen B7H1' OR 'antigen CD274' OR 'antigens, CD274' OR 'B7 H1 antigen' OR 'B7 H1 protein' OR 'B7 homolog 1 protein' OR 'B7-H1 antigen' OR 'B7H1 antigen' OR 'B7H1 protein' OR 'CD274 antigen' OR 'CD274 antigens' OR 'PDCD1 ligand 1' OR 'PDCD1LG1 protein' OR 'programmed cell death 1 ligand 1' OR 'programmed death 1 ligand 1 protein' OR 'programmed death ligand 1' OR 'protein B7 H1' OR 'protein B7H1' OR 'protein PDCD1LG1' OR 'programmed death 1 ligand 1' OR 'nivolumab' OR 'pembrolizumab' OR 'durvalumab' OR 'avelumab' OR 'atezolizumab' OR 'ipilimumab' OR 'tremelimumab' OR 'cemiplimab' OR 'camrelizumab' OR 'tislelizumab' OR 'sintilimab' OR 'toripalimab' |
|  | #2 | 'tuberculosis/exp OR 'tuberculosis' OR 'active TB' OR 'active tuberculosis' OR 'case of TB' OR 'cases of TB' OR 'chronic tuberculosis' OR 'infection by M. tuberculosis' OR 'infection by Mycobacterium tuberculosis' OR 'infection due to M. tuberculosis' OR 'infection due to Mycobacterium tuberculosis' OR 'infection of M. tuberculosis' OR 'infection of Mycobacterium tuberculosis' OR 'Koch`s disease' OR 'M. tuberculosis infection' OR 'minimal tuberculosis' OR 'minimum tuberculosis' OR 'Mycobacterium tuberculosis infection' OR 'TB (tuberculosis)' OR 'TB case' OR 'TB cases' OR 'TB disease' OR 'TB infection' OR 'tuberculous infection' OR 'tuberculous lesion' |
|  | #3 | ^#^1 AND ^#^2 |
|  | #4 | ^#^3 Filters: human |
| Cochrane | #1 | 'MeSH descriptor: [Immune Checkpoint Inhibitors] explode all trees' OR 'PD L1 Inhibitors' OR 'PD L1 Inhibitor' OR 'PD-L1 Inhibitors' OR 'Programmed Death-Ligand 1 Inhibitors' OR 'PD-L1 Inhibitor' OR 'Programmed Death Ligand 1 Inhibitors' OR 'PD 1 PD L1 Blockade' OR 'Blockade, PD-1-PD-L1' OR 'PD-1-PD-L1 Blockade' OR 'Checkpoint Blockers, Immune' OR 'Checkpoint Inhibitor, Immune' OR 'Checkpoint Inhibitors, Immune' OR 'Immune Checkpoint Inhibitor' OR 'Immune Checkpoint Blockers' OR 'CTLA-4 Inhibitor' OR 'Cytotoxic T-Lymphocyte-Associated Protein 4 Inhibitor' OR 'Cytotoxic T-Lymphocyte-Associated Protein 4 Inhibitors' OR 'CTLA 4 Inhibitor' OR 'CTLA-4 Inhibitors' OR 'CTLA 4 Inhibitors' OR 'Cytotoxic T Lymphocyte Associated Protein 4 Inhibitor' OR 'Cytotoxic T Lymphocyte Associated Protein 4 Inhibitors' OR 'PD-1 Inhibitors' OR 'Inhibitor, PD-1' OR 'Programmed Cell Death Protein 1 Inhibitor' OR 'PD 1 Inhibitor' OR 'Programmed Cell Death Protein 1 Inhibitors' OR 'PD 1 Inhibitors' OR 'PD-1 Inhibitor' OR 'Immune Checkpoint Inhibition' OR 'Checkpoint Inhibition, Immune' OR 'Immune Checkpoint Blockade' OR 'Checkpoint Blockade, Immune' OR 'nivolumab' OR 'pembrolizumab' OR 'durvalumab' OR 'avelumab' OR 'atezolizumab' OR 'tremelimumab' OR 'ipilimumab' OR 'camrelizumab' OR 'sintilimab' OR 'tislelizumab' OR 'toripalimab' |
|  | #2 | ['MeSH descriptor: [Tuberculosis] explode all trees' OR 'Kochs Disease' OR 'Tuberculoses' OR 'Koch Disease' OR 'Infection, Mycobacterium tuberculosis' OR 'Infections, Mycobacterium tuberculosis' OR 'Koch's Disease' OR 'Mycobacterium tuberculosis Infection' OR 'Mycobacterium tuberculosis Infections'](#0) |
|  | #3 | ^#^1 AND ^#^2 |
|  | #4 | ^#^3 Filters: human |

Table S2: Subgroup Analysis of TB Risk

| Subgroup | Studies (n) | ICI Group* | Non-ICI Group* | Relative Risk (95% CI) | I² (%) | Z | p value |
| --- | --- | --- | --- | --- | --- | --- | --- |
| Previous history of TB |  |  |  |  |  |  |  |
| Yes | 2^[15, 19]^ | 27 | 927 | 1.093 (0.284–4.207) | 85.2% | 0.13 | 0.897 |
| No | 3^[7, 16, 18]^ | 25 | 72 | 7.319 (1.187–45.121 | 88.2% | 2.14 | 0.001 |
| *Patients (TB events) |  |  |  |  |  |  |  |
